# Regulation of IL-8 gene expression in gliomas by microRNA miR-93

**DOI:** 10.1186/s12885-015-1659-1

**Published:** 2015-10-08

**Authors:** Enrica Fabbri, Eleonora Brognara, Giulia Montagner, Claudio Ghimenton, Albino Eccher, Cinzia Cantù, Susanna Khalil, Valentino Bezzerri, Lisa Provezza, Nicoletta Bianchi, Alessia Finotti, Monica Borgatti, Giuseppe Moretto, Marco Chilosi, Giulio Cabrini, Roberto Gambari

**Affiliations:** 1 Department of Life Sciences and Biotechnology, Section of Biochemistry and Molecular Biology, University of Ferrara, Via Fossato di Mortara n.74, 44121 Ferrara, Italy; 2 Department of Pathology and Diagnostics, Laboratory of Molecular Pathology, University-Hospital of Verona, P.le A Stefani n.1, 37126 Verona, Italy; 3 Department of Neurosciences, University-Hospital of Verona, P.le A Stefani n.1, Verona, 37126 Italy

**Keywords:** microRNA, IL-8 mRNA, Glioma

## Abstract

**Background:**

Different strategies have been proposed to target neoangiogenesis in gliomas, besides those targeting Vascular Endothelial Growth Factor (VEGF). The chemokine Interleukin-8 (IL-8) has been shown to possess both tumorigenic and proangiogenic properties. Although different pathways of induction of IL-8 gene expression have been already elucidated, few data are available on its post-transcriptional regulation in gliomas.

**Methods:**

Here we investigated the role of the microRNA miR-93 on the expression levels of IL-8 and other pro-inflammatory genes by RT-qPCR and Bio-Plex analysis. We used different disease model systems, including clinical samples from glioma patients and two glioma cell lines, U251 and T98G.

**Results:**

IL-8 and VEGF transcripts are highly expressed in low and high grade gliomas in respect to reference healthy brain; miR-93 expression is also increased and inversely correlated with transcription of IL-8 and VEGF genes. Computational analysis showed the presence of miR-93 consensus sequences in the 3′UTR region of both VEGF and IL-8 mRNAs, predicting possible interaction with miR-93 and suggesting a potential regulatory role of this microRNA. *In vitro* transfection with pre-miR-93 and antagomiR-93 inversely modulated VEGF and IL-8 gene expression and protein release when the glioma cell line U251 was considered. Similar data were obtained on IL-8 gene regulation in the other glioma cell line analyzed, T98G. The effect of pre-miR-93 and antagomiR-93 in U251 cells has been extended to the secretion of a panel of cytokines, chemokines and growth factors, which consolidated the concept of a role of miR-93 in IL-8 and VEGF gene expression and evidenced a potential regulatory role also for MCP-1 and PDGF (also involved in angiogenesis).

**Conclusion:**

In conclusion, our results suggest an increasing role of miR-93 in regulating the level of expression of several genes involved in the angiogenesis of gliomas.

**Electronic supplementary material:**

The online version of this article (doi:10.1186/s12885-015-1659-1) contains supplementary material, which is available to authorized users.

## Background

Several possible targets of therapeutic interventions against gliomas have been recently proposed, such as EGFR [1], VEGF [2], the Akt-pathway [3] and the NF-kappaB pathway [4]. In addition to these important targets, the production of cytokines and chemokines might be of interest, since these proteins have been associated to glioma invasion [5–10].

Among these proteins, interleukin-8 (IL-8, or CXCL8) is now known to be a major promoter of angiogenesis and invasiveness of human gliomas, where it is expressed and secreted at high levels [11–13]. Among the different control levels of IL-8 gene expression in gliomagenesis, several activator mechanisms have been studied and well characterized, such as hypoxia/anoxia stimulation, response to Fas ligation, death receptor activation, activity of cytosolic Ca^2+^ transients, TNF-α, IL-1, other cytokines and various cellular stresses [14]. One of the control levels is transcriptional and related to the interaction with the IL-8 promoter of different transcription factors, such as NF-kappaB, AP-1, and C-EBP/NF-IL-6 [15–18]. In addition, the expression of the IL-8 gene might be under the control of epigenetic mechanisms, such as those regulated by microRNAs in both cancer and inflammatory processes [19–24].

MicroRNAs (miRs) (www.mirbase.org) belong to a family of small (19 to 25 nucleotides in length) noncoding RNAs that target specific sequences of mRNAs thereby regulating gene expression [25, 26], with the induction of translational repression or mRNA degradation, depending on the degree of complementarities between miRs and the target sequences [27, 28]. Considering that a single miR can recognize several mRNAs and a single mRNA might contains in its sequence (3′UTR, CDS, 5′UTR) several signals for molecular recognition by miRs, it is calculated that more than 60 % of mammalian mRNAs are target of microRNAs [28], controlling metabolic pathways in differentiation, cell cycle and apoptosis [27, 28].

MiR-dependent regulation of IL-8 gene expression has been recently shown both in inflammatory [29] and in cancer [20, 30–33] experimental model systems. For instance, we found that the effects of bacterial challenge activating IL-8 gene transcription in epithelial cells, are down regulated by miR-93, which acts as a potent feedback mechanism [29]. This is of peculiar interest for cancerogenesis, since miR-93 has been found involved in the down regulation of integrin β-8 [34] and VEGF expression [35]. Besides the finding that IL-8 can be regulated by miR-155 dependent modulation of the transcription factor Interferon Regulatory Factor 3 in malignant glioma cell lines [36], little is known about miR-dependent regulation of IL-8 gene expression in gliomas.

The aim of this research was first to study the expression of microRNA miR-93 and IL-8 gene in low-grade (LGG) and high-grade gliomas (HGG) specimens *ex vivo* (a), glioma cell lines transfected with antagomiR-93 (b) and pre-miR-93 (c). Expression of miR-93 and IL-8 mRNA was analyzed by RT-qPCR and production of IL-8 was detected using Bio-plex analysis. VEGF was used as a control, since it has been reported that this is a miR-93 regulated gene [35]. Second, we wanted to compare the IL-8 results with the data obtained on other chemokines, cytokines and growth factors.

## Methods

### Human tissue samples

Human glioma specimens of deceased patients, obtained after surgery and fixed with the formalin-free alcoholic-based fixative FineFIX (Milestone SrL, Sorisole, Bergamo, Italy) and paraffin embedded, previously utilized for histological diagnosis and in the archive of the Unit of Pathology, have been obtained according to the Declaration of Helsinki and following the specific authorization of the local Ethical Committee to which the University Hospital of Verona refers (CESC - Comitato Etico Sperimentazione Clinica VR/RO - Protocol CESC VR RO 22/01/2014 - 5.1.3). Informed written consent from the patients has been obtained. Personal data have been treated according to the Italian Legislation (GU no. 72-2012/03/26 - article 4) to guarantee that each sample is anonymous. Histological diagnosis and grading has been confirmed separately by two expert pathologists (C.G. and A.E.). High-Grade Gliomas (HGG) were all grade IV glioblastomas whereas Low-Grade Gliomas (LGG) were all classified as grade II tumors, according to 2007 WHO classification [37]. Three 10 μm sections from each sample were utilized to extract RNA either for total RNA or miRNA analyses.

### Glioma cell lines and culture conditions

U251 [38] and T98G [39] cells were cultured in humidified atmosphere of 5 % CO_2_/air in RPMI 1640 medium (Life Technologies, Monza, Italy) supplemented with 10 % fetal bovine serum (FBS, Celbio, Milan, Italy), 100 U/ml penicillin and 100 mg/ml streptomycin (Sigma-Aldrich, St. Louis, USA). To verify possible effects on proliferation, cell growth was monitored by determining the cell number/ml using a Z1 Coulter Counter (Coulter Electronics, Hialeah, FL, USA).

### Expression of IL-8 and VEGF mRNA by in situ hybridization (ISH)

ISH assay was performed using the RNA scope 2.0 HD Reagent Kit Brown (cat no. 310035) with the probes for Hs-IL-8 (cat no. 310381), Hs-VEGF (cat no. 423161), Hs-GAPDH (positive control; cat no. 310321) and DapB (negative control; cat no. 310043) according to the protocol provided by Advanced Cell Diagnostics (Hayward, CA). Serial tissue sections were scanned by D-sight 2.0 System (Menarini Diagnostics, Firenze, IT).

### Pre-miR and AntagomiR transfections

U251 and T98G glioma cells were transfected with 200 nM antagomiR-93, pre-miR-93 and the miR negative controls (Ambion, Applied Biosystem, Foster City, CA, US) complexed with siPORT NeoFX (Life Technologies, Carlsbad, CA, US). After 48 h, cell supernatants were collected; total RNA was extracted and immediately converted to cDNA.

### RNA isolation

RNA to quantitate both IL-8 mRNA, VEGF mRNAs and miR-93 was extracted from formalin-free alcoholic-based fixative FineFIX and paraffin embedded samples of the archive of deceased patients by MiRNeasy FFPE minikit (Qiagen, Venlo, Limburg, Netherlands). Reference RNA from healthy brain was purchased from Clontech (Clontech Laboratories, Mountain View, CA, USA) and obtained from the whole brain of a 28-yr-old Asian male deceased because of sudden death. Mir-93 expression in LGG, HGG and healthy brain RNA samples was firstly calculated relative to U6 snRNA. Samples from LGG and HGG were subsequently expressed as Fold Changes (FC) in respect to Clontech reference RNA obtained from healthy brain tissue. Total RNA from U251 cells and T98G cells was isolated using Tri-reagent (Sigma Aldrich). The 2100 bioanalyzer was used to determine the integrity and measure the concentration of total RNA samples (Agilent Technologies, Instrument DE54700480, Eukaryote Total RNA Nano Series II.xsy).

### Quantitation of IL-8 and VEGF mRNA content

Total RNA (1 μg) was reverse-transcribed to cDNA using the High Capacity cDNA Archive Kit and random primers (Applied Biosystems). IL-8 and VEGF mRNAs analyzed with RT-qPCR were quantified by TaqMan Gene Expression Assays (Applied Biosystems, codes HS00174103m1 and HS00173626_m1), respectively, and normalized to calibrator genes GAPDH mRNA (code HS02758991_g1), RPL13A (code HS03043885_g1), 18S rRNA (code 4310893E) according to the manufacturer’s instructions, with a 7900HT Fast Real Time PCR System (Applied Biosystems). Relative quantification of gene expression was performed using the comparative threshold (C_T_) method as described by the manufacturer (Applied Biosystems User Bulletin 2). Changes in mRNA expression level were expressed as fold change over untreated samples.

### Quantitation of miR-93

Quantitation of miR-93 was performed by specific reverse transcription and TaqMan probes with TaqMan MicroRNA Assays (Applied Biosystems, code 00432). MiR-93 expression was firstly normalized to U6 snRNA (code 001973) and let-7c (code 000379). Mir-93 expression in LGG, HGG and healthy brain RNA sample, firstly calculated relative to U6 snRNA, was subsequently expressed as Fold Changes (FC) in respect to reference RNA from the Clontech healthy brain tissue.

### Bio-Plex-analysis

Cytokines, chemokines and growth factors in tissue culture supernatants released from the cells under analysis, were measured by Bio-Plex Pro Human Cytokine 27-plex Assay (#M50-0KCAF0Y, Bio-Rad Laboratories, Hercules, CA) as described by the manufacturer [40, 41]. The Bio-Plex cytokine assay is designed for the multiplexed quantitative measurement of multiple cytokines in a single well using as little as 50 μl of sample. Samples were analyzed on a Bio-Rad 96-well plate reader using the Bio-Plex Suspension Array System and Bio-Plex Manager software (Bio-Rad Laboratories, Hercules, CA) [40, 41].

### Statistics

Results were expressed as mean ± standard deviation (S.D.). Comparisons between groups were made by using paired or unpaired Student’s *t* test for *in vitro* or *ex vivo* analyses, respectively. Statistical significance was defined with *p* < 0.05 (statistically significant, *) and *p* < 0.01 (highly statistically significant, **).

## Results

### In situ hybridization reveals expression of VEGF and IL-8 mRNA in glioma tissues

**Fig. 1:**
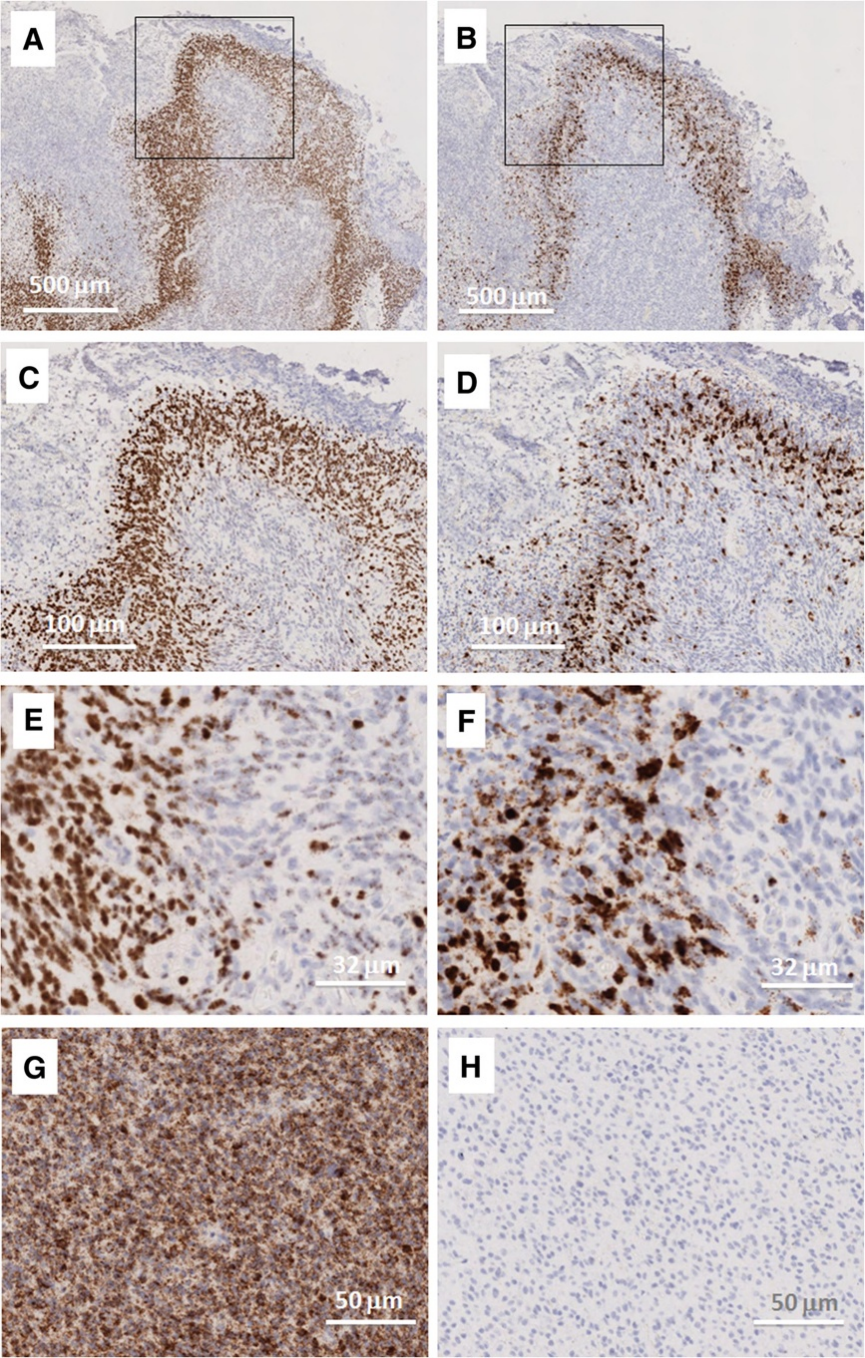
Expression of IL-8 and VEGF mRNA in glioblastoma. VEGF mRNA (**a**, **c**, **e**) and IL-8 mRNA (**b**, **d**, **f**) by mRNA *in situ* hybridization are shown in separate 5 μm serial tissue sections from glioblastoma specimens at different magnifications (**a**, **b**: x2; **c**, **d**: x10; **e**, **f**: x32) by peroxidase staining. Nuclei are counterstained with hematoxylin. Positive (GAPDH mRNA) and negative (DAPB mRNA) controls are reported (**g**, **h**: x20 magnification). Squared areas in panels A and B indicate the detail reported in panels **c** and **d**, respectively

Expression of IL-8 and VEGF mRNA in glioblastoma tissues was studies by *in situ* hybridization performed in separate 5 mm serial tissue sections from glioblastoma specimens. Figure 1 shows that VEGF and IL-8 mRNAs are expressed at very high levels in the same histological areas of the glioma.

Staining of IL-8 mRNA was observed in similar areas of the glioma that express VEGF. Interestingly, the IL-8 and VEGF staining was found mainly associated with the areas showing hypoxic features and most frequently in those astrocytic spindle cells characterizing the “pseudopalizading” pattern, observed in the areas in proximity to hypoxia and hypoxic necrosis, which represents a histological hallmark of the glioblastoma. This supports previously published evidences from different laboratories pointing out that both VEGF and IL-8 are markers of glioma progression, linked to late stages of development and neoangiogenic processes induced by hypoxia. Therefore, RT-qPCR analysis was performed on tissue specimens obtained from patients with low-grade and high-grade gliomas.

### Expression of VEGF and IL-8 in patients with low-grade and high-grade gliomas

**Fig. 2:**
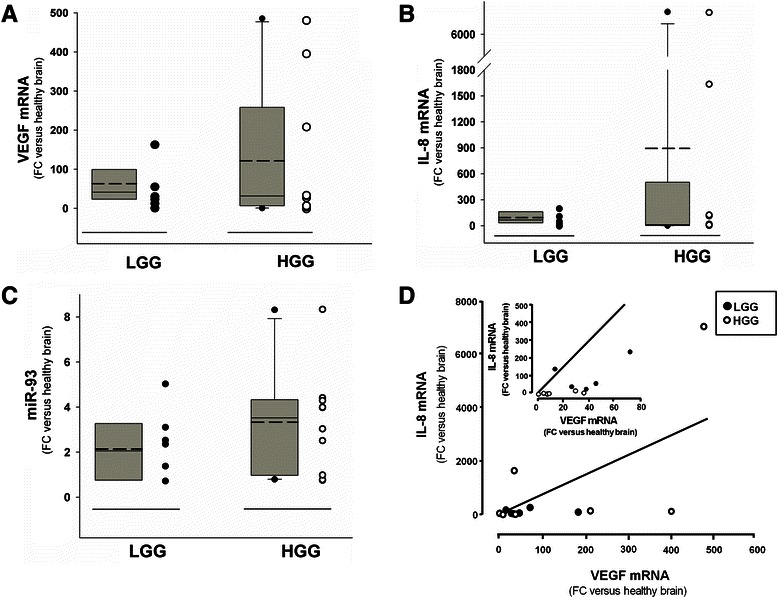
Expression of VEGF, IL-8 and miR-93 in Low-Grade Gliomas (LGGs) and High-Grade Gliomas (HGGs). VEGF mRNA (**a**) and IL-8 mRNA (**b**) levels relative to GAPDH were measured by RT-qPCR with TaqMan probes on RNAs isolated from FFPE sections of 6 LGG and 10 HGG and normalized to healthy brain reference RNA. Fold changes (FC) of expression over healthy brain reference RNA are reported. In the same LGGs and HGGs miR-93 was quantified (**c**) and normalized to healthy brain reference RNA. For panels **a**–**c**: dashed line: mean; solid line: median; grey box includes values from 5th to 95th centiles, vertical lines range from min to max values, excluding outliers which are represented by single dots. The data obtained in each glioma specimen are reported in the right side of the panels. **d** Relationship between VEGF mRNA and IL-8 mRNA in the same LGG (filled circles) and HGG (open circles) samples analyzed and reported in **a**–**c**. Regression straight line showing direct correlation was drawn by the least square method Sigmaplot. Inset reports the same graph expanded

We verified the IL-8 expression in low-grade glioma (LGG) and high-grade glioma (HGG) tissues, since IL-8 expression has been related to the process of tumor neoangiogenesis, a hallmark of transition from low to high grade gliomas. We used VEGF as a comparison, since VEGF is a validated marker of neoangiogenesis in gliomas and, more important within the framework of this study, it is a validated target of miR-93 [35]. In Fig. 2 (a and b) the expression of VEGF and IL-8 genes in LGG and HGG is shown in comparison with the expression levels of reference healthy brain tissues. RNA was extracted from tissue sections and analyzed by RT-qPCR. Both VEGF (Fig. 2a) and IL-8 (Fig. 2b) mRNAs are up-regulated in LGG in respect to reference healthy brain. It should be noted that IL-8 and VEGF are even further up-regulated in many HGGs in respect to LGGs (Fig. 2a and b), confirming the striking genetic heterogeneity which characterizes HGGs. Interestingly, a positive correlation trend between IL-8 and VEGF expression levels can be observed in most of the different LGG and HGG cases (Fig. 2d), strongly suggesting a co-regulation of VEGF and IL-8 genes in HGG. The level of expression of miR-93 reported in Fig. 2c indicates an up-regulation in LGG and HGG in comparison to the expression measured in reference healthy brain. Also in the case of miR-93, its levels of expression are more heterogeneous in HGG samples, prompting us to verify a possible correlation of its expression levels with those of VEGF and IL-8.

### IL-8 is a putative target of miR-93 in gliomas

**Fig. 3:**
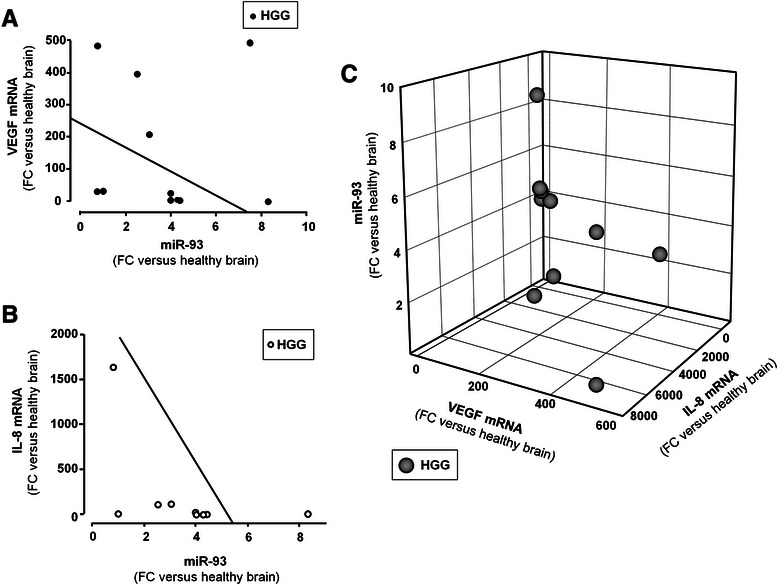
Correlations among the expression of miR-93, VEGF and IL-8 mRNAs in HGGs. Regression analysis between VEGF mRNA (**a**) and IL-8 mRNA (**b**) was performed as function of miR-93. Regression straight line showing inverse correlation was drawn by the least square method with Sigmaplot software. The graphical correlations among miR-93, VEGF and IL-8 mRNAs is represented in 3D plot (**c**). All data are reported as fold changes (FC) over healthy brain reference RNA

**Fig. 4:**
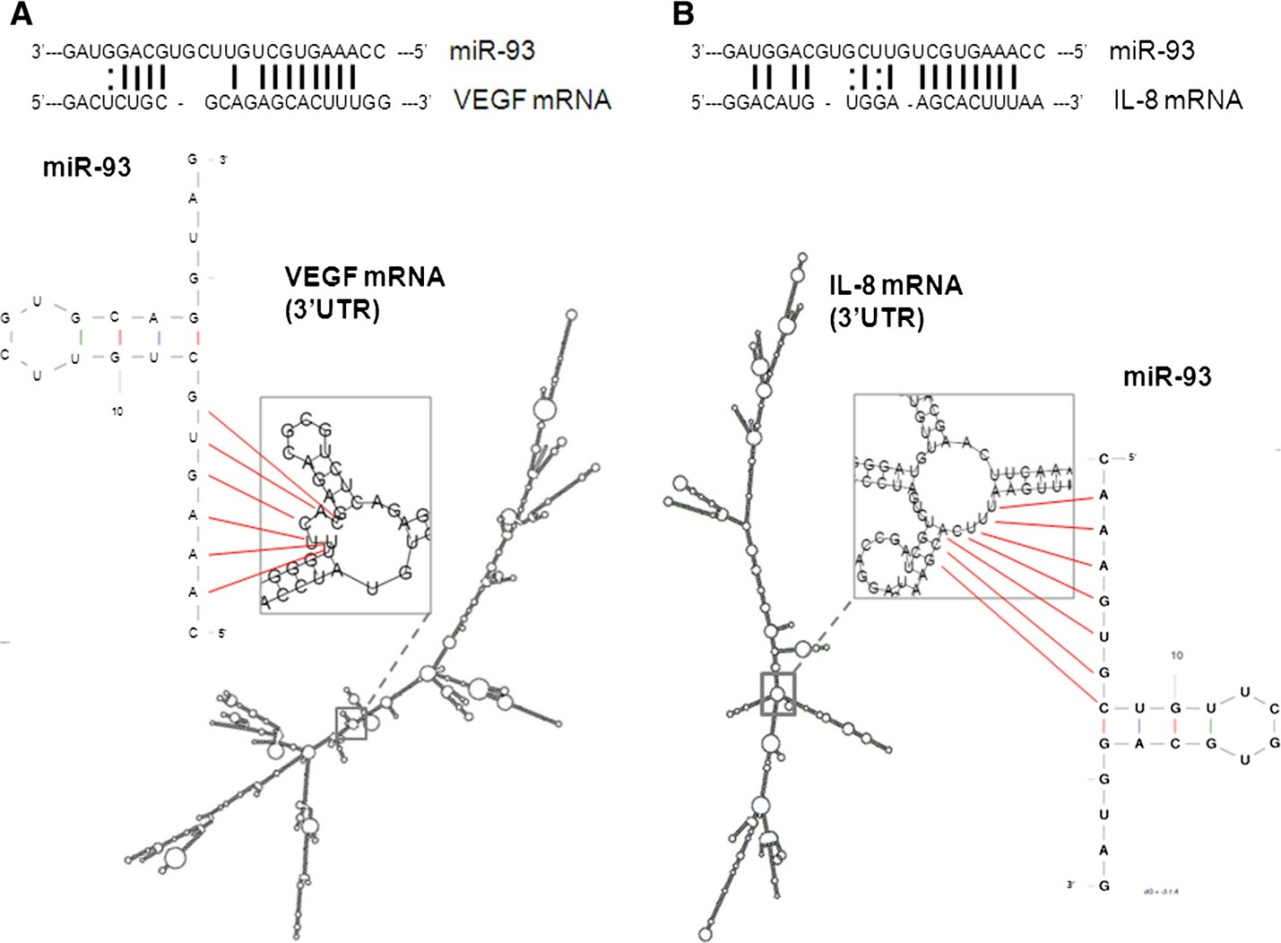
Interactions of miR-93 with IL-8 mRNA and VEGF mRNA. Predicted secondary structure of the 3′UTR regions of VEGF mRNA (**a**) and IL-8 mRNA (**b**) based on the UCSC genome browser (http://genome.ucsc.edu), and of miR-93 by UNAFold Web Server (http://mfold.rna.albany.edu). Magnification is also shown of the central portion of 3′UTR IL-8 (**b**) and VEGF (**a**) mRNAs and pointing out the possible interaction between the 3′UTR target strands and the seed region of the lowest energy miR-93 potential stem loops

The inverse correlation between miR-93 levels and VEGF and IL-8 expression is of relevance, as shown in Fig. 3, since these two genes might be under the post-transcriptional control of miR-93, as recently proposed in different experimental model systems [21, 29, 35]. Figure 4 reports the possible interactions between miR-93 and miR-93 binding sites located within the 3′UTR sequence of VEGF mRNA and IL-8 mRNA. The miR-93 target sequences of VEGF and IL-8 mRNAs are shown, indicating possible base-pairing with miR-93. These predicted analyses support the hypothesis that both VEGF and IL-8 mRNAs are target of miR-93.

### Correlation of the expression of miR-93 with VEGF and IL-8 mRNA levels

When the results of the analysis of miR-93 expression in HGG is correlated with that of VEGF (Fig. 3a) and IL-8 (Fig. 3b) mRNAs, an inverse correlation can be found in most cases (i.e. high levels of miR-93 are present in HGG samples with low VEGF and IL-8 mRNA content and, vice versa, high VEGF and IL-8 mRNA content are present when expression of miR-93 is lower). This conclusion is supported by the comprehensive 3D analysis shown in Fig. 3c, where the parallel decrease of IL-8 and VEGF mRNAs expression is associated with increased levels of miR-93. Therefore, in order to experimentally verify the hypothesis that miR-93 is involved in the regulation of both IL-8 and VEGF, we modulated the miR-93 expression in the human glioma cell line U251 by transfecting the cells with pre-miR-93 and antagomiR-93 molecules.

### Treatment of U251 glioma cells with antagomiR-93 and pre-miR-93: effects on VEGF secretion

**Fig. 5:**
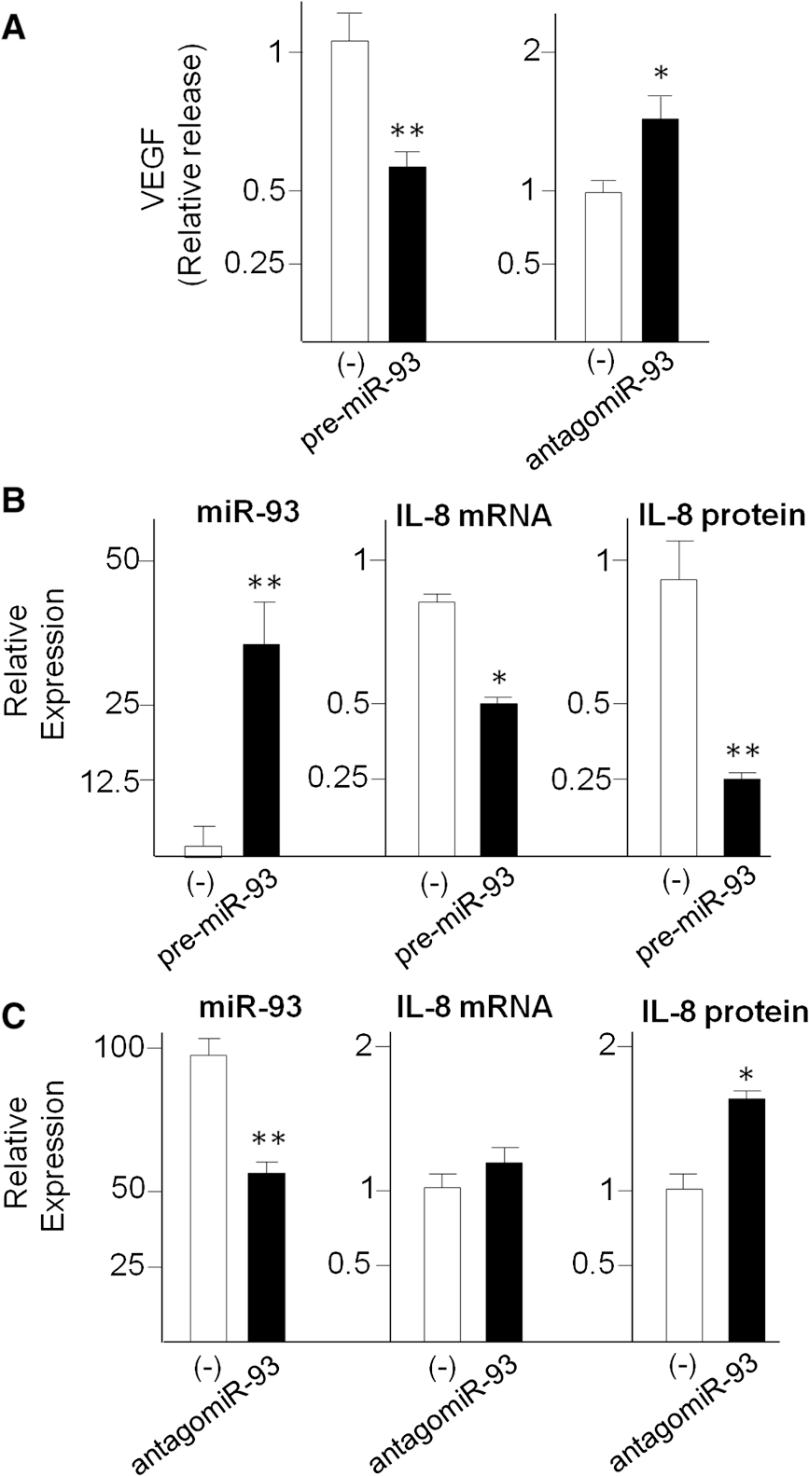
Effects of the treatments of glioma U251 cells with pre-miR-93 and antagomiR-93. VEGF (**a**) and IL-8 released protein (**b**, **c**) were quantified by Bio-plex analysis. RNA was isolated from cultures after 48 h *in vitro* growth and analyzed by RT-qPCR. Internal RT-qPCR control were U6 snRNA and let-7c for miR-93, RPL13A and 18S for IL-8 mRNA. Data are in all cases reported in comparison to U251 cells treated with control scrambled sequences. Results represent the average ± S.D. of at least three independent experiments. * *p* < 0.05; ** *p* < 0.01

Figure 5a shows a first set of experiments in which pre-miR-93 and antagomiR-93 have been transfected for 48 h into U251 cells and the secretion of VEGF protein was determined. VEGF secretion was analyzed by Bio-Plex assay. 200 nM pre-miR-93 and antagomiR-93 were administrated with the transfection reagent. The results of Fig. 5a demonstrate that a sharp decrease of released VEGF was found when U251 glioma cells were transfected with a pre-miR-93 RNA (left side). The results reported in Fig. 5a, (right side), demonstrate increase of VEGF release in cells in which down-regulation of miR-93 was forced by transfection with antagomiR-93. These data show that the validated miR-93 target VEGF is modulated as expected in U251 glioma cells transfected with pre-miR-93 and antagomiR-93 molecules.

### Reduction of IL-8 gene expression in U251 glioma cells transfected with pre-miR-93

Figure 5b shows experiments in which pre-miR-93 has been transfected to U251 cells and IL-8 gene expression was determined by RT-qPCR and Bio-plex analysis of released protein. The results reported demonstrate that when U251 glioma cells are transfected with a pre-miR-93 RNA, (a) the level of miR-93 sequences, as expected, increases (Fig. 5b, left panel) and (b) a decrease of IL-8 mRNA occurs (Fig. 5b, central panel). This is confirmed by Bio-Plex analysis performed using the cell growth medium, in which a sharp decrease of released IL-8 protein was found in pre-miR-93 treated U251 cells (Fig. 5b, right panel).

### AntagomiR-93 stimulates increase of IL-8 expression in U251 glioma cells

The use of antagomiR sequences to target microRNA might also help in understanding the involvement of these sequences in biological functions as published in several reports [42, 43]. In this context, we determined whether treatment of the U251 glioma cell line with antagomiR against miR-93 led to induction of IL-8. To this aim, U251 glioma cells were transfected with antagomiR-93 and the expression of miR-93 analyzed by RT-qPCR. In addition, IL-8 mRNA content and IL-8 secretion were analyzed by RT-qPCR and Bio-Plex assays, respectively. AntagomiR-93 was administrated at the concentration of 200 nM with the siPORT NeoFX transfection reagent. The results reported in Fig. 5c (left panel) demonstrate that antagomiR-93 reduces the miR-93 accumulation in U251 glioma cells. Figure 5c demonstrates that the forced down-regulation of miR-93 is accompanied by a slight increase of IL-8 mRNA (Fig. 5c, central panel) and a significantly higher release of IL-8 (Fig. 5c, right panel), fully in agreement with the hypothesis of an involvement of miR-93 in IL-8 gene expression.

### Transfection with pre-miR-93 and antagomiR-93 alters IL-8 gene expression in glioma cell lines U251 and T98G

**Fig. 6:**
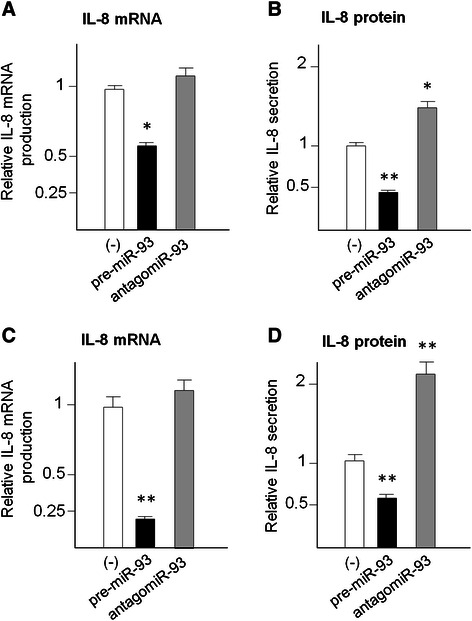
Effects on IL-8 mRNA (**a**, **c**) and IL-8 protein (**b**, **d**) of the treatments of glioma U251 (**a**, **b**) and T98G (**c**, **d**) cells with pre-miR-93 and antagomiR-93. RNA was isolated from cultures after 48 h *in vitro* growth and analyzed by RT-qPCR. Internal RT-qPCR control was RPL13A. Released IL-8 protein was quantified by Bio-plex analysis. Data are in all cases reported in comparison to U251 and T98G cells treated with control sequences. Results represent the average ± S.D. of three independent experiments. * *p* < 0.05; ** *p* < 0.01

Figure 6 shows that the modulation of IL-8 gene expression is similar in two different glioma cell lines (U251 and T98G) treated as described in Fig. 5 with pre-miR-93 and antagomiR-93 sequences. When the two glioma cell lines were treated with pre-miR-93, a sharp decrease of IL-8 mRNA accumulation and IL-8 secretion was observed. When transfection with antagomiR-93 was performed, no major differences were found in comparison to untreated cells; however, in both U251 and T98G cell lines, a significant increase of IL-8 secretion was found. Therefore, we concluded that the miR-93 dependent regulation of IL-8 gene expression is operated in both the glioma cell lines investigated. In parallel with the experiments reported in Figs. 5 and 6, treated U251 and T98G cells were analyzed also for cellular morphology and possible induction of apoptosis, obtaining consistent and highly reproducible data demonstrating the lack of not specific or toxic effects of these treatments, as reported in Additional file 1: Figure S1.

### Modulation of miR-93 expression confirms its predominant role in IL-8 post-transcriptional regulation

**Fig. 7:**
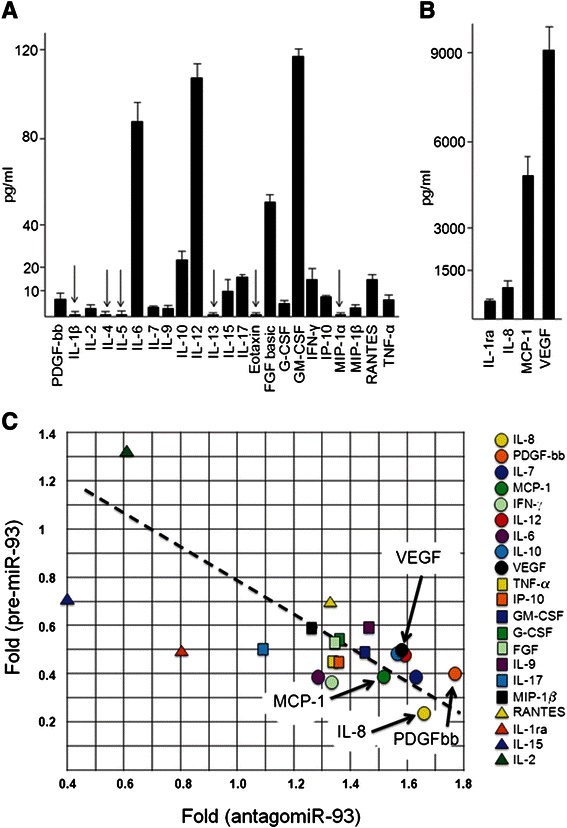
Secretome profile of U251 cells. **a**, **b** Arrowed are protein exhibiting low level of secretion by U251 cells. **c** Changes in the protein profile after treating U251 cells with antagomiR-93 and pre-miR-93. The data generating this panel are shown in Table 1 and are originated by Bio-plex analysis

In order to verify whether miR-93 selectively regulates IL-8 gene expression within a group of other pro-inflammatory genes, a 27-plex cytokine assay was carried on in the supernatants collected from glioma U251 cells cultured in the absence or in the presence of antagomiR-93 or pre-miR-93. Figure 7 (a and b) shows the secretome of U251 cells, demonstrating a strong difference with respect to protein release. Proteins released with highest efficiency were IL-8, MCP-1 and VEGF (Fig. 7b). This confirms data published in other studies [44–47]; moreover the high release of these proteins were confirmed following analysis after 48 and 72 h of cell culture, as shown in Additional file 1: Figure S2. Proteins released with very low efficiency (IL-1β, IL-4, IL-5, IL-13, Eotaxin, MIP-1α, below 2.5 pg/ml) are arrowed in panel A of Fig. 7; these were excluded from our analysis. In some cases we found a relevant inverse correlation between fold increase of secretion following antagomiR-93 treatment (leading as shown in Figs. 5 and 6 to miR-93 down regulation) and relative content of secreted proteins in cells pre-transfected with pre-miR-93 (Fig. 7b). We applied an algorithm for determining the miR-93 dependency index (miR-93_INDEX_) of U251 cells, based on the determination of the treated/untreated fold values and which is as follows: fold(pre-miR-93 treatment)/fold(antagomiR-93 treatment). Following this algorithm we expect low values of miR-93_INDEX_ for those genes whose expression in regulated by miR-93. These data generating the miR-93_INDEX_ are shown in Fig. 7c. The miR-93_INDEX_ values for the different cytokines/chemokines/growth factors are indicated in Table 1. Taken together, these results strongly suggest that IL-8 gene displays the highest levels of sensitivity to miR-93. Besides IL-8, the other genes showing miR-93 dependency higher than or similar to VEGF (used as reference gene in consideration of its already demonstrated dependency from miR-93 activity) were PDGF-bb, GM-CSF, MCP-1, IFN-γ, IL-12, IL-6 and IL-10. Interestingly several of them (IL-8, VEGF, PDGF-bb, MCP-1) are demonstrated to play a significant role in the late stage of glioma progression, including interaction with the microenvironment leading to angiogenesis [2–8, 12–14].

**Table 1:** U251 secretome after treatment with pre-miR-93 or antagomiR-93

Protein	Release (pg/ml)	Fold release after pre-miR-93 treatment	Fold release after antagomiR-93 treatment	miR-93 dependency (miR-93_INDEX_)^a^
PDGF-bb	5.96	0.40	1.76	0.23
IL-1ra	475.02	0.49	0.80	0.61
IL-2	3.84	1.32	0.62	2.10
IL-6	85.8	0.39	1.28	0.30
IL-7	4.17	0.38	1.64	0.23
IL-8	838.38	0.27	1.63	0.17
IL-9	3.53	0.59	1.47	0.40
IL-10	25.15	0.48	1.57	0.30
IL-12	105.99	0.46	1.59	0.29
IL-15	9.60	0.72	0.40	1.80
IL-17	14.80	0.50	1.09	0.47
FGF	49.55	0.52	1.33	0.39
G-CSF	6.30	0.53	1.37	0.39
GM-CSF	114.50	0.49	1.44	0.34
IFN-γ	14.47	0.36	1.32	0.27
IP-10	7.80	0.45	1.37	0.33
MCP-1(MCAF)	4743.32	0.38	1.51	0.25
MIP-1β	2.35	0.59	1.27	0.47
RANTES	13.69	0.68	1.32	0.51
TNF-α	8.51	0.43	1.32	0.33
VEGF	9469.12	0.49	1.56	0.31

^a^miR-93 dependency: fold (pre-miR-93 treatment)/fold (antagomiR-93 treatment)

## Discussion

The first conclusion of this paper is that the microRNA miR-93 is involved in the control of the expression of the IL-8 gene in the glioma U251 cell line on the basis of the effects of parallel transfections with pre-miR-93 or antagomiR-93.

The effects of these treatments were analyzed by RT-qPCR (looking at the IL-8 mRNA content) or by Bio-plex analysis (looking at IL-8 protein secretion). The data obtained allow to suggest that miR-93 is involved in the regulation of IL-8 gene expression in gliomas, in agreement with already reported results supporting the concept that IL-8 mRNA is a true miR-93 molecular target [21, 29, 35]. We also analyzed the effect of the pre-miR-93 and antagomiR-93 treatments on the secretome in multiplexing analysis conducted on 27 cytokines/chemokines/growth factors. Preliminarily, we analyzed the overall secretion, excluding those proteins for which low secretion (>2.5 pg/ml) was found. The data obtained are shown in Table 1 and Fig. 6, which demonstrates that miR-93 dependency is particularly evident for IL-8 (index: 0.17). Interestingly, the results presented in Fig. 7 and Table 1 suggest that other genes known to be involved in glioma invasion and angiogenesis (arrowed in Fig. 7c) together with IL-8, could be post-transcriptionally regulated by miR-93, including for instance MCP-1 (index: 0.25), IL-6 (index: 0.30), PDGF-bb (index: 0.23), as well as VEGF, which was already demonstrated to be regulated by miR-93 in gliomas (index: 0.31). As expected from Table 1, we found miR-93 binding sites in MCP-1, IL-6 and PDGF-bb mRNAs, in agreement with the found miR-93_INDEX_. On the contrary, no miR-93 binding sites were found within the 3′-UTR sequences of IL-2 and IL-15 mRNAs, strongly supporting the hypothesis that these mRNAs are not regulated by this microRNA, as also suggested by the data reported in Table 1.

These data support the concept that miR-93 down regulation might lead to up-regulation of genes involved in the HGG phenotype. In this respect we found that HGG samples are very heterogeneous in IL-8 expression and miR-93 content (see Fig. 2b), which is expected due to the high genetic variability of glioblastomas [48], which includes also the variable expression of microRNAs [49]. In spite of this wide genetic heterogeneity, an inverse relationship between IL-8 mRNA and miR-93 can be observed in our samples of glioblastomas (see Fig. 3b), similar to that found between VEGF mRNA and miR-93 (see Fig. 3a), as evidenced also by plotting both IL-8 and VEGF mRNAs in respect to miR-93 (see Fig. 3c). This indicates that, although the post-transcriptional control of the expression of IL-8 gene is in principle dependent on the interaction of multiple microRNAs, the relevance of miR-93 in the control of IL-8 gene expression in glioblastoma is gaining ground.

We found a three to eight fold increased expression levels of miR-93 in our glioma specimens in respect to healthy brain reference samples, as shown in Fig. 2c, with higher expression in HGG versus LGG, which opens the question of the role of miR-93 in glioma initiation and progression. Increased expression of miR-93 could be consistent with its oncogenic role as proposed in other cancers, since miR-93 has been shown to down-regulate the cell cycle inhibitor p21, impairing the TGFβ − mediated cell cycle arrest [50]. Of great interest is the recent report by Codo et al. [51] showing that high miR-93 expression in gliomas might be related to down-modulation of NKG2DL, one glioma-associated ligand interacting with one of the major activating receptors of natural killer (NK) cells. This miR-93 mediated NKG2DL leads to reduced susceptibility of tumor glioma cells to NK-mediated lysis [51]. In addition to this biological effect, overexpression of miR-93 has been shown to promote tumor growth and angiogenesis by suppressing, at least in part, integrin-β8 expression in the U98 astrocytoma cell model [34]. In contrast to these “pro-oncogenic roles” of miR-93, here we found that miR-93 down-regulates the expression of two well-established pro-angiogenic genes such as VEGF and IL-8, as shown in Figs. 3 and 5, which fits more likely with a tumor suppressing role. Considering together Codo’s [51], Fang’s [34] and our findings, we could speculate on a differential role of miR-93 as a function of its expression levels and/or the stage of progression of the gliomas.

As far as the expression of IL-8 gene is concerned, we propose that the increase of IL-8 gene expression from healthy brain to LGG is not caused by a decrease of miR-93 expression, but by other regulatory network associated with IL-8 gene transcription. Among these, the NF-kappaB network should be carefully considered for the following reasons: (a) NF-kappaB is one of the major transcription factors involved in IL-8 gene regulation [18]; (b) NF-kappaB is a marker of glioma onset and progression [12, 13, 52, 53]; (c) miR-16 inhibits glioma cell growth through suppression of the NF-kappaB signaling pathway [54].

In respect of this speculation, it has been shown that miR-93 plays a dual role in malignant breast stem cells, acting as a tumor promoter in normal breast stem cells and, on the opposite, as a tumor suppressor in poorly differentiated malignant breast stem cells [55], suggesting the need to further analyze the role of miR-93 in different stages of progression of gliomagenesis.

**Fig. 8:**
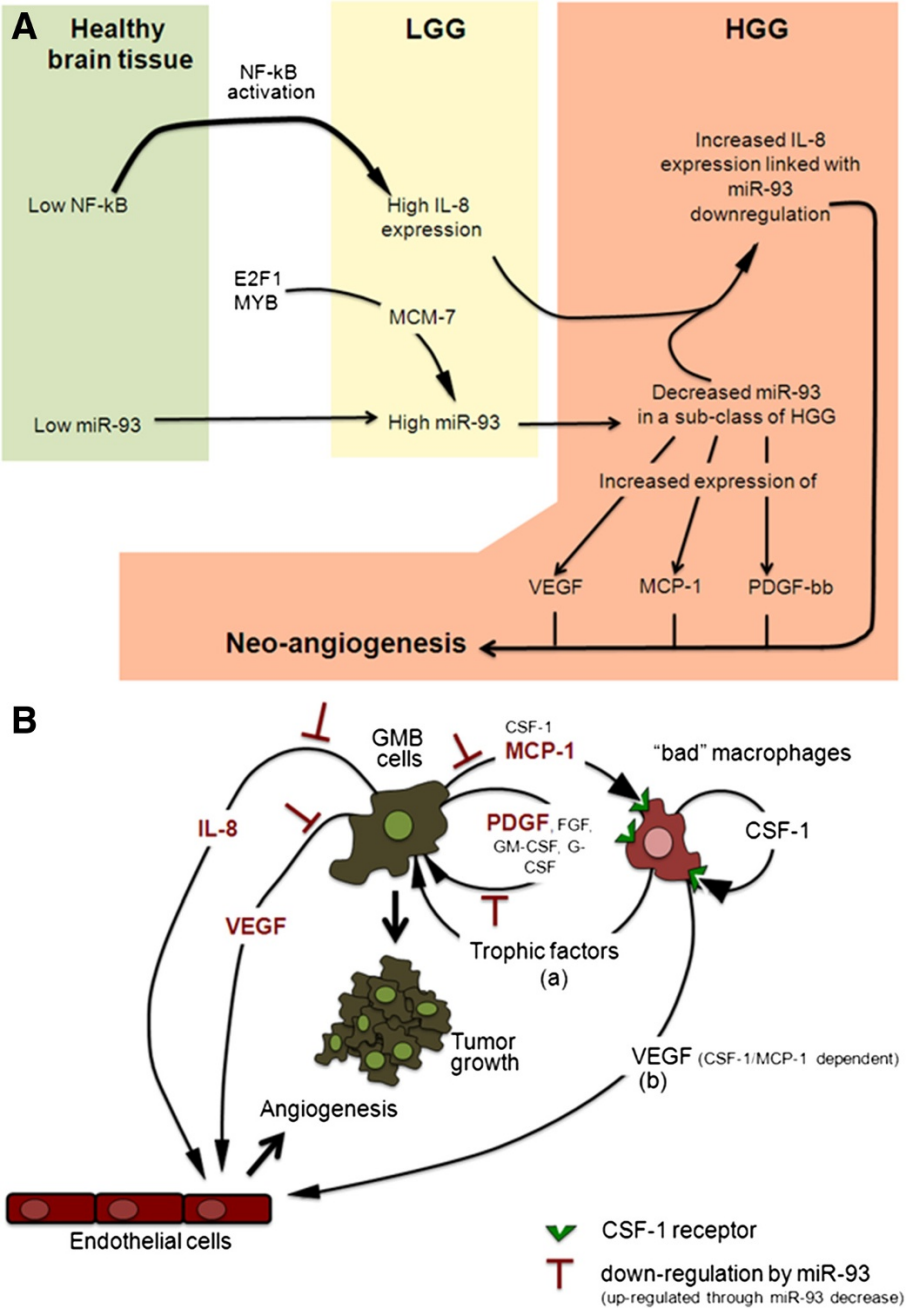
**a** Proposed model for the control of the changes of IL-8 and miR-93 expression during the transition from normal brain tissues to LGG and to HGG. In the scheme is also indicated some effects (namely of VEGF, MCP-1, PDGF-B) associated to miR-93 down-regulation during LGG to HGG transition. **b** Proposed inhibitory effects of miR-93 within the microenvironment participating to glioma angiogenesis [59]

In conclusion, the data reported in this paper sustain the concept that miR-93 is able to regulate IL-8 gene expression, as it was found recently in other cellular systems [29]. However, as shows in the scheme of Fig. 8a and elsewhere suggested [15–18], the increase of IL-8 expression in LGG in respect to normal brain tissues is not caused by decrease of miR-93 but by other mechanisms of action, possibly including NF-kappaB activation. In this respect it is well known that NF-kappaB is one of the most important transcription factors regulating IL-8 gene expression [18] and it was widely reported to be highly expressed in glioma, being associated with promotion of growth and angiogenesis [13]. Furthermore, NF-kappaB activation might be operated in glioblastomas through the oncogenic effects of miR-196, leading to inhibition of IkappaBα (and therefore NF-kappaB activation) both in vitro and in vivo [56]. The further increased IL-8 gene expression in HGG (with respect to LGG) might be on the contrary associated with decrease of its inhibitory microRNA miR-93, at least in a sub-set of HGG patients, on the basis of the analysis of the clinical samples shown in Figs. 2 and 3. The decrease of miR-93 in these HGG patients, in addition to IL-8, might lead to a post-transcriptional up-regulation of VEGF, MCP-1 and PDGF-bb, well recognized markers of late tumor stages of gliomas [34, 57, 58]. However, it should be noted that our study strongly support the concept that the HGG samples are highly heterogeneous with respect to miR-93 levels, suggesting multiple regulatory pathway in controlling the level of IL-8 gene expression. Further experiments will be necessary to verify whether other microRNAs are involved in IL-8 gene regulation.

In the HGG cases in which miR-93 is down-regulated, our data might be of interest when considered in respect to possible involvement of the down-regulation of this microRNA in controlling microenvironment by glioma cells. This is depicted in Fig. 8b which summarizes the putative cross-talk between glioma cells, macrophages and endothelial cells, following a elsewhere reported scheme [59]. The data presented in our paper suggest indeed that miR-93 down-regulation might be a key factor in sustaining the autocrine loop controlled by GM-CSF, FGF, G-CSF and the miR-93 regulated PDGF-bb. On the other hand, down-regulation of miR-93 might increase IL-8 and VEGF releases, inducing endothelial cells to support angiogenesis. VEGF can be also released by “bad” macrophages stimulated by miR-93 regulated MCP-1 [59].

In addition to basic science implications, our data might be of interest in applied biomedicine, since we demonstrate that forced expression of miR-93 is able to reduce IL-8 gene expression; therefore, molecules mimicking pre-miR-93 activity might be proposed to reduce IL-8 dependent angiogenesis in gliomas.

## Conclusion

Our results suggest a role of miR-93 in regulating the level of expression of several genes involved in the angiogenesis of gliomas. In particular miR-93 was confirmed to control the expression of VEGF and proposed to play a key role in regulating IL-8 mRNA.

## Additional file

Additional file 1: Figure S1. Effects of pre-miR-93 and antagomiR-93 on morphology and apoptosis. A. Morphology of U251 glioma cells treated for 24 h with control, pre-miR-93 and antagomiR-93 molecules (200 nM). B,C. Effects of the different treatments on apoptosis on U251 and T98G glioma cell lines, as indicated. Apoptosis was analyzed by the Annexin-V release test [60] (B) or by caspase-3/7 production [61] (C). In panel B, the effects of a positive antagomiR-221 is also shown (see Brognara et al. (2014) [62]. Data represent the average S.D. of three independent experiments. ** = *p* < 0.01. (−): untreated cellular samples. **Figure S2.** Release of IL-8, VEGF, and MCP-1 by U251 glioma cells. U251 glioma cells were cultured for 48 and 72 h and protein release quantified by Bio-plex analysis. Data represent the average S.D. of three independent experiments. * = *p* < 0.05; ** = *p* < 0.01. Additional methods. Apoptosis was analyzed on U251 and T98G glioma cell lines after 48 h of treatment with pre-miR-93 or antagomiR-93 (200 nM). Cells were washed with sterile PBS (Phosphatebuffered Saline) and then tested with the Muse Annexin V Dead Cell kit (Millipore Corporation, Billerica, MA, USA) or Muse Caspase 3/7 kit (Millipore) [63]. The assays were performed with Muse Instrument (Millipore) [63], according to the instructions provided by the manufacturer. (PDF 360 kb)

## Abbreviations

miR: microRNA
LGG: Low-grade glioma
HGG: High-grade glioma
IL: Interleukin
VEGF: Vascular endothelial growth factor
PDGF-B: Platelet Derived Growth Factor
VCAM-1: Vascular cell adhesion molecule 1
PCR: Polymerase-chain reaction
RT: Reverse transcription
RT-qPCR: RT-quantitative PCR
