# Supplementary material for: Regulation of IL-8 gene expression in gliomas by microRNA miR-93
Source: BMC Cancer. 2015 Oct 8;15:661. doi: 10.1186/s12885-015-1659-1 (PMC4598972; doi:10.1186/s12885-015-1659-1)
Supplement: Additional file 1: Figure S1. — Effects of pre-miR-93 and antagomiR-93 on morphology and apoptosis. A. Morphology of U251 glioma cells treated for 24 h with control, pre-miR-93 and antagomiR-93 molecules (200 nM). B,C. Effects of the different treatments on apoptosis on U251 and T98G glioma cell lines, as indicated. Apoptosis was analyzed by the Annexin-V release test [60] (B) or by caspase-3/7 production [61] (C). In panel B, the effects of a positive antagomiR-221 is also shown (see Brognara et al. (2014) [62]. Data represent the average S.D. of three independent experiments. ** = p < 0.01. (−): untreated cellular samples. Figure S2. Release of IL-8, VEGF, and MCP-1 by U251 glioma cells. U251 glioma cells were cultured for 48 and 72 h and protein release quantified by Bio-plex analysis. Data represent the average S.D. of three independent experiments. * = p < 0.05; ** = p < 0.01. Additional methods. Apoptosis was analyzed on U251 and T98G glioma cell lines after 48 h of treatment with pre-miR-93 or antagomiR-93 (200 nM). Cells were washed with sterile PBS (Phosphatebuffered Saline) and then tested with the Muse Annexin V Dead Cell kit (Millipore Corporation, Billerica, MA, USA) or Muse Caspase 3/7 kit (Millipore) [63]. The assays were performed with Muse Instrument (Millipore) [63], according to the instructions provided by the manufacturer. (PDF 360 kb) [file 12885_2015_1659_MOESM1_ESM.pdf]

Regulation of IL-8 gene expression in gliomas by microRNA miR-93

Additional File

Enrica Fabbri[1], Eleonora Brognara[1], Giulia Montagner[1], Claudio Ghimenton[2], Albino Eccher[2], Cinzia Cantù[3], Susanna Khalil[3], Valentino Bezzerri[3], Lisa Provezza[2], Nicoletta Bianchi[1], Alessia Finotti[1], Monica Borgatti[1], Giuseppe Moretto[3], Marco Chilosi[2], Giulio Cabrini[2] and Roberto Gambari[1]

[1]Department of Life Sciences and Biotechnology, Ferrara, Italy; [2]Department of Pathology and Diagnostics, University-Hospital, Verona, Italy [3]Department of Neurosciences, University-Hospital, Verona, Italy.

Corresponding authors:

*Roberto Gambari,
Department of Life Sciences and Biotechnology,
University of Ferrara, Via Fossato di Mortara, 74
44121 Ferrara, Italy
e-mail address: gam@unife.it;
phone: +39-0532-974443; fax: +39-0532-974500

*Giulio Cabrini,
Department of Pathology and Diagnostics,
Laboratory of Molecular Pathology,
University-Hospital, Verona, P.le A Stefani n.1
37126 Verona, Italy
e-mail address: giulio.cabrini@univr.it;
phone: +39-045-8122457, fax: +39-045-8122840

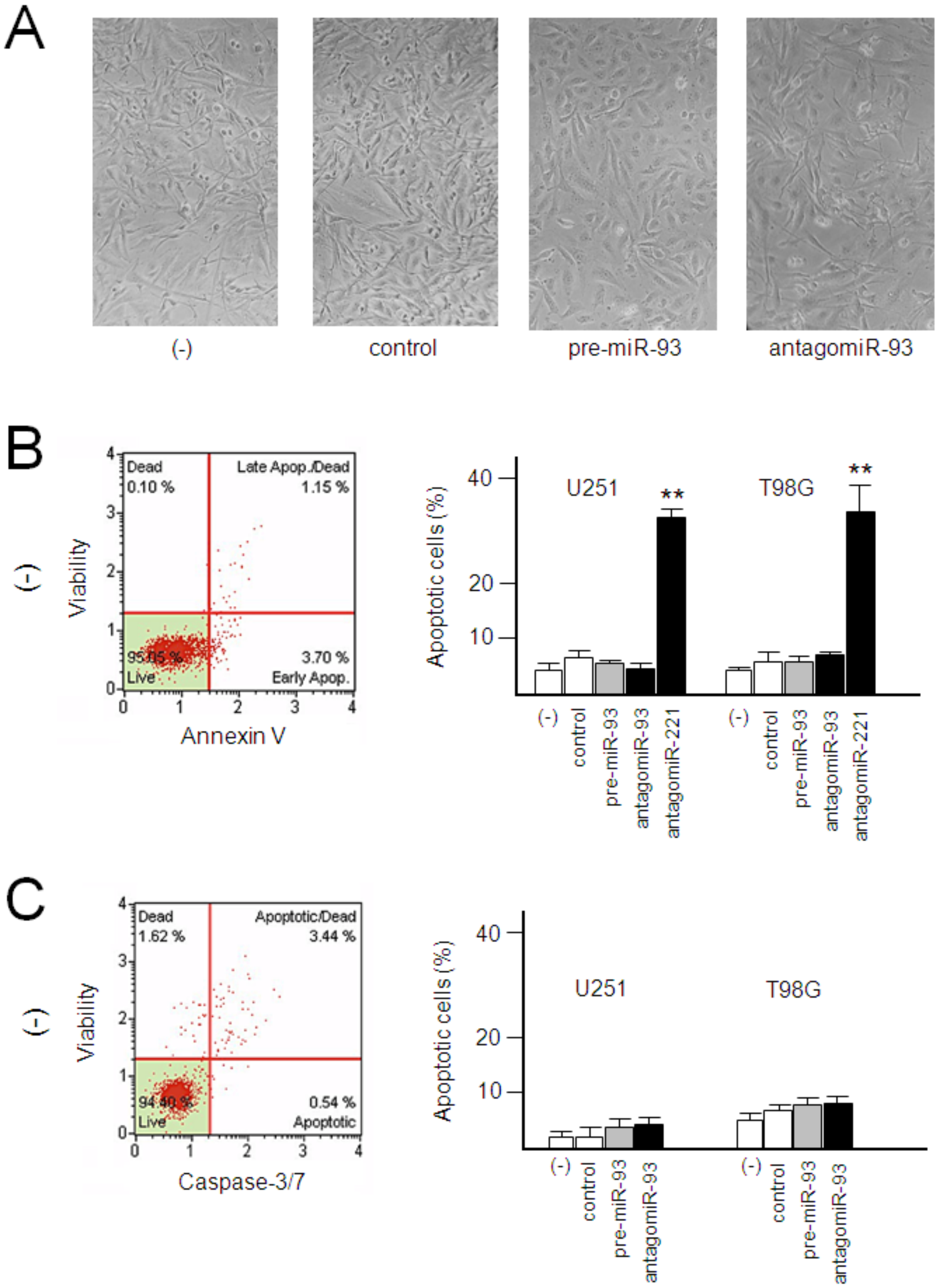

Additional File Figure 1. A. Morphology of U251 glioma cells treated for 24 hours with control, pre-miR-93 and antagomiR-93 molecules (200 nM). B,C. Effects of the different treatments on apoptosis on U251 and T98G glioma cell lines, as indicated. Apoptosis was analyzed by the annexin-V release test [1] (B) or by caspase-3/7 production [2] (C). In panel B, the effects of a positive antagomiR-221 is also shown (see Brognara et al., 2014)[3]. Data represent the average S.D. of three independent experiments. ** = p<0.01. (-): untreated cellular samples.

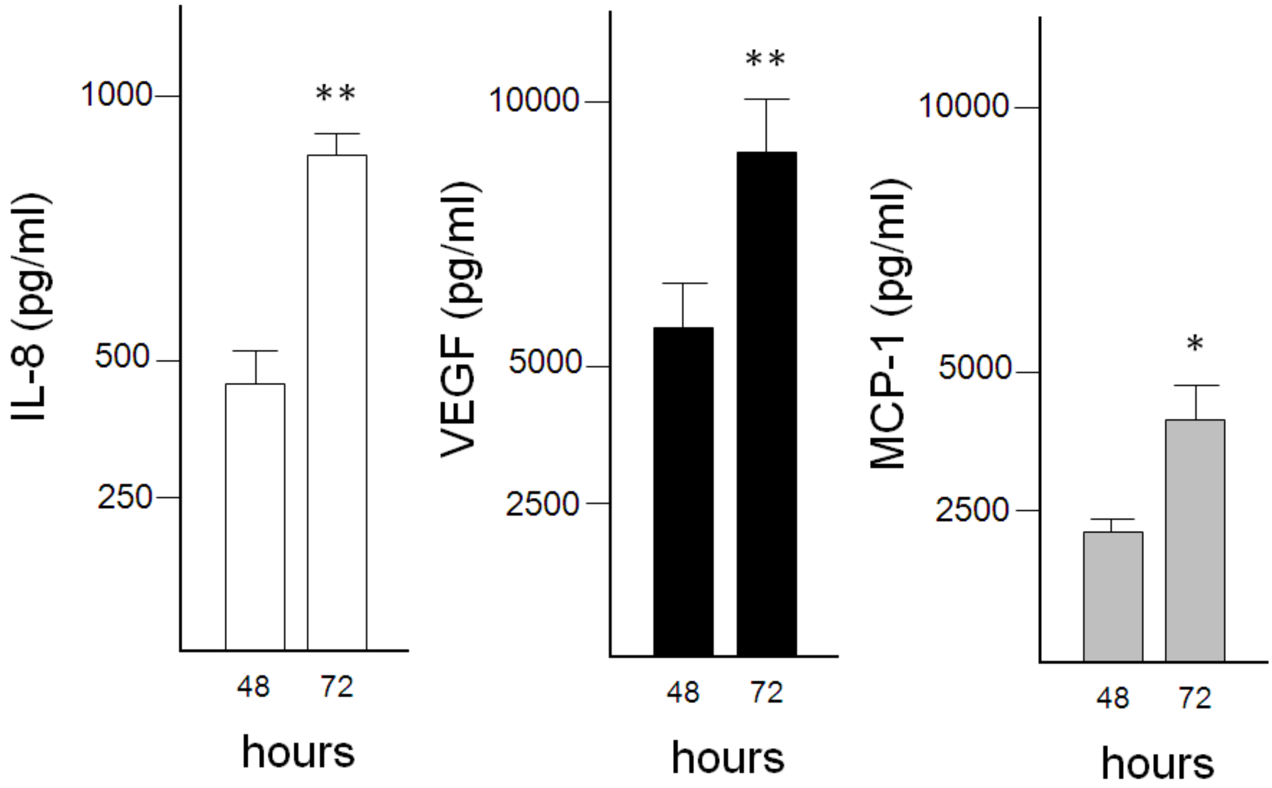

Additional File Figure 2. Release of IL-8, VEGF and MCP-1 by U251 glioma cells cultured for 48 and 72 hours. Protein release was quantified by Bio-plex analysis. Data represent the average S.D. of three independent experiments. * = p<0.05; ** = p<0.01.

ADDITIONAL METHODS

Apoptosis was analyzed on U251 and T98G glioma cell lines after 48h of treatment with pre-miR-93 or antagomiR-93 (200nM). Cells were washed with sterile PBS (Phosphate-buffered Saline) and then tested with the Muse Annexin V Dead Cell kit (Millipore Corporation, Billerica, MA, USA) or Muse Caspase 3/7 kit (Millipore) [4]. The assays were performed with Muse Instrument (Millipore) [4], according to the instructions provided by the manufacturer.

ADDITIONAL REFERENCES

[1] van Engeland M, Nieland LJ, Ramaekers FC, Schutte B, Reutelingsperger CP. **Annexin V-affinity assay: a review on an apoptosis detection system based on phosphatidylserine exposure.** *Cytometry*.1998; 31:1-9.

[2] McIlwain DR, Berger T, Mak TW. **Caspase Functions in Cell Death and Disease.** *Cold Spring Harb Perspect Biol* 2013;5:a008656

[3] Brognara E, Fabbri E, Bazzoli E, Montagner G, Ghimenton C, Eccher A, Cantù C, Manicardi A, Bianchi N, Finotti A, Breveglieri G, Borgatti M, Corradini R, Bezzerri V, Cabrini G, Gambari R. **Uptake by human glioma cell lines and biological effects of a peptide-nucleic acids targeting miR-221.** *J Neurooncol*. 2014; 118:19-28.

[4] Aboud OA, Wettersten HI, Weiss RH. **Inhibition of PPARα Induces Cell Cycle Arrest and Apoptosis, and Synergizes with Glycolysis Inhibition in Kidney Cancer Cells.** *Plosone* 2013
